# *inlF* Enhances *Listeria monocytogenes* Early-Stage Infection by Inhibiting the Inflammatory Response

**DOI:** 10.3389/fcimb.2021.748461

**Published:** 2022-02-10

**Authors:** Zhiting Ling, Dan Zhao, Xinyu Xie, Hao Yao, Yuting Wang, Suwei Kong, Xiang Chen, Zhiming Pan, Xin’an Jiao, Yuelan Yin

**Affiliations:** ^1^ Jangsu Key Laboratory of Zoonosis, Yangzhou University, Yangzhou, China; ^2^ Key Laboratory of Prevention and Control of Biological Hazard Factors (Animal Origin) for Agrifood Safety and Quality, The Ministry of Agriculture and Rural Affairs of the People’s Republic of China, Yangzhou University, Yangzhou, China; ^3^ Joint International Research Laboratory of Agriculture and Agri-Product Safety of the Ministry of Education of China, Yangzhou University, Yangzhou, China; ^4^ Jiangsu Co-innovation Center for the Prevention and Control of Important Animal Infectious Disease and Zoonosis, Yangzhou University, Yangzhou, China

**Keywords:** *Listeria monocytogenes*, *inlF*, serovar 4b, immune evasion, inflammatory injury, colonization

## Abstract

The internalin family proteins, which carry the leucine repeat region structural motif, play diverse roles in *Listeria monocytogenes* (Lm) infection and pathogenesis. Although Internalin F, encoded by *inlF*, was identified more than 20 years ago, its role in the Lm anti-inflammatory response remains unknown. Lm serotype 4b isolates are associated with the majority of listeriosis outbreaks, but the function of *InlF* in these strains is not fully understood. In this study, we aimed to elucidate the role of *inlF* in modulating the inflammatory response and pathogenesis of the 4b strain Lm NTSN. Strikingly, although *inlF* was highly expressed at the transcriptional level during infection of five non-phagocytic cell types, it was not involved in adherence or invasion. Conversely, inlF did contributed to Lm adhesion and invasion of macrophages, and dramatically suppressed the expression of pro-inflammatory cytokines interleukin (IL)-1β and tumor necrosis factor (TNF-α). Consistent with the *in vitro* results, during Lm infection mice, *inlF* significantly inhibited the expression of IL-1β and IL-6 in the spleen, as well as IL-1β, IL-6, and TNF-α in the liver. More importantly, *inlF* contributed to Lm colonization in the spleen, liver, and ileum during the early stage of mouse infection *via* intragastric administration, inducing severe inflammatory injury and histopathologic changes in the late stage. To our knowledge, this is the first report to demonstrate that *inlF* mediates the inhibition of the pro-inflammatory response and contributes to the colonization and survival of Lm during the early stage of infection in mice. Our research partly explains the high pathogenicity of serovar 4b strains and will lead to new insights into the pathogenesis and immune evasion of Lm.

## Introduction

*Listeria monocytogenes* (Lm) is a ubiquitous foodborne pathogen with worldwide distribution, comprising of 14 serotypes that, are determined by the serological reaction between specific surface carbohydrate (O-) antigens and flagella (H-) antigens (Yin et al., 2019). Of the 14 serovars identified in the Lm species, serovars 1/2a, 1/2b, and 4b strains are responsible for over 95% isolations from clinical cases of human listeriosis, among which, 4b strains alone account for 49% of *Listeria*-related foodborne diseases (Goulet et al., 2006). Chen et al. reported that the risk factor of 4b strains causing listeriosis was 100-fold higher than that of 1/2a and 1/2c strains (Chen et al., 2007). Thus far, invasive 4b strains are responsible for economic losses in the animal husbandry industry and threaten human health. Therefore, unveiling the interaction between invasive 4b strain and host can better understand the pathogenic mechanism and contribute to control *Listeria* infection. Whereas, previous studies have mostly dealt with the identification and functional characterization of bacterial cell surface-associated virulence proteins in the Lm serovar group 1/2.

Surface proteins retained by the bacterial cell wall play a critical role in Lm infection and pathogenesis. Of particular importance are the internalin family proteins, which contain leucine-rich repeats (LRRs) that mediate entry into non-phagocytic cells, allowing membrane translocation and systemic infection. Two well-known internalin proteins InlA and InlB are required for bacterial adhesion and internalization. InlA interaction with its human host receptor E-cadherin mediates the crossing of the intestinal barrier (Sahu et al., 2007). InlB triggers bacterial entry by interacting with the hepatocyte growth factor receptor (Met), gC1q-R, and proteoglycans (Jonquières et al., 2001), thus contributing to Lm colonization in the liver and spleen (Lecuit et al., 2004). InlC is involved in the adhesion and invasion of microvascular endothelial cells as well as in the weakening of the innate immune response. InlJ is exclusively expressed *in vivo* during animal infection, where it interacts with mucin secreted by intestinal epithelial cells and contributes to the virulence of Lm (Sabet et al., 2005; Sabet et al., 2008). InlH is not involved in adhesion and invasion, but can interfere with the host immune response and contribute to immune evasion (Schubert et al., 2001). InlK protein can interact specifically with the fornix protein (MVP) in the cytoplasm to mediate bacterial autophagy escape in a manner that does not cross-react with ActA actin polymerization (Dortet et al., 2011). InlP is a newly discovered, secreted internalin protein that is important for listeriosis and has strong tropism for the placenta (Faralla et al., 2016). Recently, increasing attention has been paid to the integral role of InlF in regulating a series of processes related to infection. Although many members of the internalin family of virulence factors have been discovered, their functional elucidation is only the tip of an iceberg, with the mechanisms of Lm infection and host immune evasion still poorly understood.

Once Lm has colonized the gastrointestinal tract, immediate immune responses are essential for the clearance of these bacteria. An innate immune response is required to defend against early Lm infection ahead of the T-cell-mediated immune responses mounted to eradicate the intracellular bacteria (Unanue, 1997; Pamer, 2004; Stavru et al., 2011). Cytokines play a crucial role in the host’s activation of the innate immune system (Zharkova et al., 2017). It mainly includes the pro-inflammatory cytokine tumor necrosis factor (TNF)-α, which has an important role in inducing the T helper type 1 (Th1) immune response against cellular pathogens (Allie et al., 2013; Romagnoli et al., 2017). Interleukin (IL)-6 is a key cytokine in the control of inflammation, promoting T -cell differentiation, regulating the cellular immune response, and clearing intracellular Lm (Hoge et al., 2013). Recent studies have highlighted multiple strategies by which Lm evades innate defenses. For instance, N-deacetylation of the peptidoglycan in the bacterial cell wall helps Lm to avoid recognition by Toll-like receptor 2 and NOD-like receptors NOD1 and NOD2, thus escaping *via* manipulation of the host’s autophagic machinery (Boneca et al., 2007). Another survival strategy involves the Lm virulence factor listeriolysin O, which uses a mitochondrial sequence NOD-like receptor NLRX1 to hijack the host cell’s mitotic homeostasis system (Zhang Y. et al., 2019). Although there are couples of reports about *inlF*, its role in the inflammatory response has not yet been reported, further efforts are required to bridge this knowledge gap.

In this study, we investigated the role of *inlF* in the infection and pathogenesis of Lm serovar 4b strain NTSN, which was isolated from an ovine outbreak. We compared the transcriptional expression characteristics *inlF* and other internalin genes, examined the adhesion and invasion abilities of *inlF* in the wild type, deletion and complemented strain in five non-phagocytic cells and macrophages, and used *in vitro* and *in vivo* models to unravel the role of *inlF* in the modulation of the host innate immune response. To our knowledge, these results represent the first report of *inlF*-mediated interference with the innate immune response and contribution to Lm survival during the early stage of infection.

## Materials and Methods

### Bacterial Strains and Cell Lines

We isolated the virulent Lm strain NTSN from a case of ovine listeriosis. The *Escherichia coli* DH5α strain and pGEM-T and pAULA vectors were obtained from the Jiangsu Key Laboratory of Zoonosis, and the shuttle vector pKSV7 was kindly donated by Professor Zhu Guoqiang (Yangzhou University, China). The cell lines used in this study are listed in **Supplementary Table 1** and were propagated in Dulbecco’s modified Eagle medium (Gibco, USA) supplemented with 10% fetal bovine serum (CLARK, USA).

### Animals and Ethics Statement

Six-week-old female C57BL/6 and BALB/c mice were purchased from the Vital River Laboratory Animal Technology Co., Ltd. (Beijing, China). Animal experiments were conducted following the guidelines laid down for the welfare and ethics of experimental animals. All animals were kept at the animal biosafety facilities and the study was conducted in accordance with procedures approved by the Institutional Animal Ethics Committee of Yangzhou University.

### Analysis of Gene Expression at the Transcriptional Level

Total RNA from mouse tissues or bacterial cultures was extracted using an RNeasy Plus Mini Kit (Qiagen, Germany). The mRNA in mouse liver or spleen was collected using an RNA prep pure animal tissues Extraction Kit (Tiangen, China). Synthesis of cDNA was performed with a PrimeScript™ RT reagent kit protocol (Takara, China) using 1 µg total RNA. Primers used for quantitative real-time PCR (qRT-PCR) are listed in **Supplementary Table 3**. The qRT-PCR operation program followed the recommended thermal cycling conditions for the FastStart Universal SYBR Green Master reaction mix (Rox, Germany). To objectively reflect differences in gene expression, *gyrb* and *gapdh* were selected as internal reference genes. The 2^ΔΔCt^ (ΔCt = Ct*_objective gene_* − Ct*_reference gene_*) method was used to calculate the relative changes in expression.

### Construction of the Mutant and Complemented Strain

To achieve homologous recombination, a plasmid carrying the *inlF* gene flanking regions *inlF*a and *inlF*b (pGEM-T-*inlF*ab) was constructed by amplifying *inlF*a and *inlF*b by PCR using primers (*inlF*a-F/*inlF*a-R and *inlF*b-F/*inl*b-R) designed from the Lm NTSN genome sequence. Oligonucleotide primer sequences are provided in **Supplementary Table 4**. A plasmid carrying *inlF*ab-(pKSV7-*inlF*ab) was positively identified, introduced into a competent culture of Lm NTSN by electroporation in accordance with a previously described protocol (Park and Stewart, 1990), and cultured in an incubator at 30°C. Positive clones were screened by *inlF*W1/*inlF*W2 and the obtained mutant was named NTSNΔ*inlF.*

To obtain the complemented strain NTSNΔ*inlF::inlF*, a plasmid carrying *inlF*cd-(pAULA-*inlF*cd) was constructed using primers *inlF*c-F/*inlF*c-R and *inlF*d-F/*inl*d-R as described above, then electroporated into NTSNΔ*inlF* competent cells. Positive clones were screened by *inlF*W3/*inlF*W4 on brain heart infusion (BHI) plates containing 5 mg/L erythromycin (Sigma, USA) at 42°C.

All constructed vectors were verified by sequencing. The cDNA isolated from Lm NTSN, NTSNΔ*inlF* and NTSNΔ*inlF::inlF* was used as template for RT-PCR with primers *inlF1/inlF2* to determine the deletion and reversion of *inlF*.

### Analysis of Biological Characteristics of Bacterial Strains

Bacterial from exponentially growing cultures of Lm NTSN, NTSN Δ *inlF* and NTSNΔ *inlF::inlF* were harvested and the optical density at 600 nm (OD_600_) was measured and adjusted to 0.05. Three parallel replicates were set for each strain. The OD_600_ value of each flask was measured at 1.5-h intervals.

Lm NTSN and NTSNΔ*inlF* were scraped from plates using an inoculating loop and transferred into 5 ml of 0.45% normal saline. The bacterial turbidity was controlled at ∼1.0 with a nephelometer and biochemical characteristics were identified using a VITEK^®^ 2 GN card (Biomérieux, France).

### Determination of Lm Invasiveness

To compare the invasive capacities of Lm NTSN, NTSNΔ*inlF* and NTSNΔ*inlF::inlF*, five non-phagocytic cell lines (Caco-2 human intestinal epithelial cells, HepG2 human hepatocytes, Hela human cervical cancer cells, BRL3A murine hepatocytes, and NIH3T3 murine fibroblasts) and the RAW264.7 macrophage cell line passaged under stabilized growth conditions were seeded into 24-well cell culture plates and cultured for 12–16 h to form monolayers with ~90% confluence. Lm NTSN, NTSNΔ*inlF* and NTSNΔ*inlF::inlF* cultures were transferred to 10 ml fresh BHI broth (1:50 ratio) and grown at 37°C to an OD_600_ value of 0.8. Cell monolayers (5 × 10^5^ cells) were infected with bacteria at a multiplicity of infection (MOI) of 20 for 1 h, after which the medium was removed and the cells were washed twice with phosphate-buffered saline (PBS). This was followed by the addition of 1 ml DMEM medium containing 100 μg/ml gentamicin sulfate, and incubation for 1 h to assess invasion ability (Yin et al., 2019). Cells were subsequently washed twice with PBS, 0.2% Triton X-100 was added and incubated for 9 min at room temperature to induce cell lysis. Serial dilutions of lysates in PBS were plated onto agar for 20 h of incubation at 37°C before determination of bacterial numbers. The bacterial invasion rate was calculated with the following formula: (number of bacteria internalized/number of initial bacteria in wells) × 100%.

### Assessment of Bacterial Loads and Inflammatory Factor Levels

Six-week-old female C57BL/6 mice were used to estimate organ colonization properties following intra-gastric inoculation with 1 × 10^9^ colony-forming units (CFU) of Lm. Mice were given 15 mg/300 µl CaCO_3_ by gavage 30 min before the bacterial inoculation to neutralize gastric acid. Lm NTSN, NTSNΔ*inlF* and NTSNΔ*inlF::inlF* (n = 7/group) were evaluated at 6, 24 and 72 h post infection by determining bacterial loads in the ileum, spleen, and liver of mice. The ileum samples were treated as follows: a 2-centimeter-length was taken and washed with 5 ml PBS by syringe to remove the luminal contents (Yin et al., 2019). The ileum was homogenized, and serial dilutions of the organ homogenates were plated onto improved oxford medium (BD Diagnostics, USA) foundation plates. Serial dilutions of livers and spleens homogenates were plated onto BHI plate.

For the determination of inflammatory cytokines, 6-week-old C57BL/6 female mice were randomly divided into three groups and inoculated abdominally with Lm NTSN, NTSNΔ*inlF* or NTSNΔ*inlF::inlF* (100 μl) at a dose of 2 × 10^5^ CFU. The spleen and liver of each mouse were harvested at 6 h post infection and used to extract total RNA. The transcriptional expression of cytokines IL-6, IL-10, TNF-α, and IL-1β was determined by qRT-PCR performed according to the specific steps described in *Analysis of Gene Expression at the Transcriptional Level* above.

### Histopathologic Analysis

Six-week-old BALB/c mice were dissected at 72 h post-intragastric inoculation with Lm at a dose of 1 × 10^9^ CFU. Following the bacterial load assessment, samples of the spleen, liver, ileum and brain were separated and fixed in 12% formaldehyde to prepare histopathologic sections. The fixed sections were subjected to hematoxylin and eosin staining at WuXi AppTec Co., Ltd.

## Results

### *inlF* Transcription Upregulated by Lm Infection *In vitro* and *In vivo*

**Figure 1:**
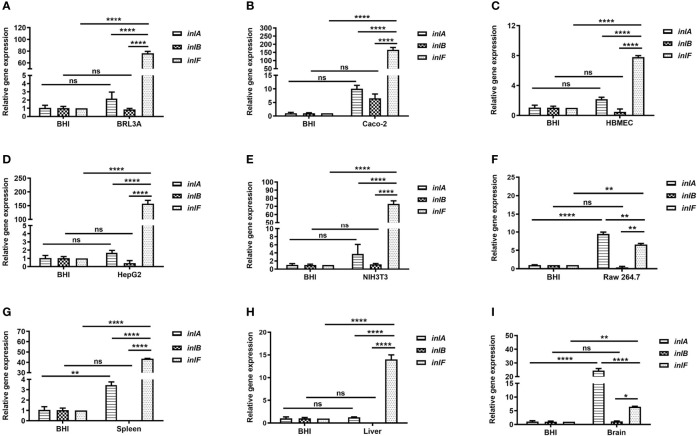
Analysis of the transcriptional expression of internalin genes. Internalins production during Lm NTSN invasion of BRL3A **(A)**, Caco-2 **(B)**, HBMEC **(C)**, HepG2 **(D)**, NIH3T3 **(E)** cells was determined by qRT-PCR. The detailed description of these cells is shown in **Supplementary Table 1**. Internalins expression in Lm NTSN infection of mice spleen, liver and brain is shown in **(F-H)**, respectively. In the experiment, six-week-old BALB/c mice were intragastrically infused at a dose of 1 × 10^9^ CFU. After 72 h, RNA was extracted from the liver, spleen and brain. BHI refers to each internalin gene’s expression under the *in vitro* culture condition. Error bars represented SEM, n = 3 independent experiments. Statistical analysis was carried out by Tukey’s multiple comparisons test: *P < 0.05, **P < 0.01, ****P < 0.0001, ns, no significance.

To understand the expression features of *inlF*, *inlA*, and *inlB* during Lm infection of multiple cell types (**Supplementary Table 1**), the mRNA transcriptional levels of each were determined and compared. Interestingly, the expression of *inlF* at the transcriptional level was higher than that of *inlA* and *inlB* (*p <*0.0001) during Lm infection of five non-phagocytic cell types, and was higher than *inlB* (*p <*0.01) during Lm invasion of RAW264.7 cells (**Figures 1A–F**). These results indicated that *inlF* may play an important role in the process of Lm invasion of various types of cells.

Lm crosses the intestinal barrier for dissemination to the organs by the blood circulation, and can also breach the blood–brain or placental barriers (Zheng and Xun, 2013). To further evaluate the role of internalins in the process of Lm infection *in vivo*, the expression of *inlF*, *inlA*, and *inlB* in the spleen, liver and brain of infected mice was measured using RT-PCR. The expression of *inlF* in the three organs was dramatically upregulated compared with Lm cultured in BHI, and its transcriptional level was significantly higher than those of *inlA* and *inlB* (*p <*0.0001) in liver and spleen (**Figures 1G, H**). In brain tissues, the transcriptional level of *inlF* was significantly higher than that of *inlB* (*p <0.05*), but significantly lower than that of *inlA* (*p <0.0001*) (**Figure 1I**). These differences in expression of *inlF*, *inlA*, and *inlB* suggested that they may play different roles in Lm invasion of different organs, with a possible involvement of *inlF* in the liver, spleen, and brain.

### Successful Construction of Mutant Lm Strains

The equivalent comparison between mutant and wild strain can provide a breakthrough for the interpretation of experimental phenomena. To decipher the role of *inlF* in Lm serovar 4b infection, we compared the biological and immunological characteristics of an *inlF* deletion strain, the parental strain and the complemented strain using *in vitro* and *in vivo* models. We constructed a deletion mutant strain (NTSN*ΔinlF)* and its reverse strain (NTSNΔ*inlF::inlF)*, with verification of the genomic knockout of *inlF* shown by RT-PCR with no DNA amplification of products with the length of 184 bp (**Supplementary Figure 1A**). The deletion of *inlF* was further confirmed by Western blotting showing the absence of an 88kDa-band in the component of the cell wall (**Supplementary Figure 1B**). Taken together, these results confirmed the successful construction of the NTSNΔ*inlF* mutant strain *via* homologous recombination technology.

### Growth and Metabolism of Lm Unaffected by Knockout of *inlF*

The growth features of the parental strain, *inlF*-deficient and reverse strains were compared by measuring growth curves. All three Lm strains cultured at 37°C exhibited similar growth trends, with logarithmic growth within 6 h, followed by entry into a stationary phase (**Supplementary Figure 2**). The growth curves verified that the deletion of *inlF* did not affect the growth of Lm in culture. The biochemical characteristics of each strain were determined by an automatic microbial identification instrument used to evaluate a potential effect of *inlF* deletion on *Listeria* metabolism. The results suggested that *inlF* was not involved in Lm NTSN physiologic or biochemical reactions of Lm NTSN (**Supplementary Table 2**).

### Involvement of *inlF* in Lm Infection Macrophages

**Figure 2:**
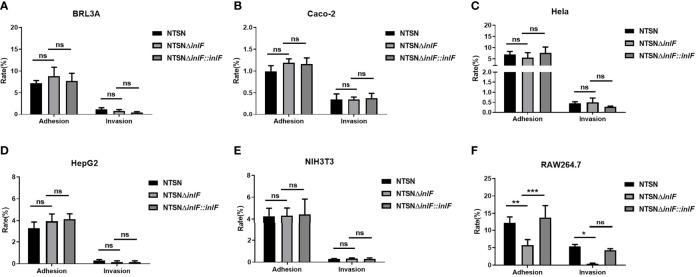
Comparative invasive capacities of Lm NTSN NTSNΔ*inlF* and NTSNΔ*inlF::inlF*. Six types of cells (**Supplementary Table 1**) were infected (MOI = 20) with Lm NTSN, NTSNΔ*inlF*, and NTSNΔ*inlF::inlF*, respectively. The percentage of intracellular bacterium was calculated after 1h of bacteria invasion. Shows are the adhesion and invasion of Lm to 5 types of non-phagocytes **(A–E)**, to macrophage RAW264.7 is shown in **(F)**. Among them, the PBS negative control group is represented by horizontal line bar, NTSN is represented by black and white check bar and the mutant is represented by black dot bar. Error bars represented SEM, n = 3 independent experiments. Statistical analysis was carried out by Tukey’s multiple comparisons test: *P < 0.05, **P < 0.01, ***P < 0.001, ns, no significance.

Testing the bacterial infection properties of a variety of different cell types is a widely used approach that offers convenience, good repeatability and a short experimental cycle. To evaluate the role of *inlF* in virulence, we examined the adhesion and invasion abilities of Lm NTSN, NTSNΔ*inlF*, and NTSNΔ*inlF::inlF* in five non-phagocytic cell types and macrophages RAW264.7 (**Supplementary Table 1**). The results showed that *inlF* was not required for *Listeria* adhesion or invasion of five non-phagocytic cell types *in vitro* (**Figures  2A–E**). By contrast, the deletion of *inlF* significantly decreased the Lm adhesion and invasion capability in RAW264.7 (*p <*0.05) (**Figure 2F**). These results indicated that *inlF* may contribute to Lm infection *via* interaction with macrophages.

**Figure 3:**
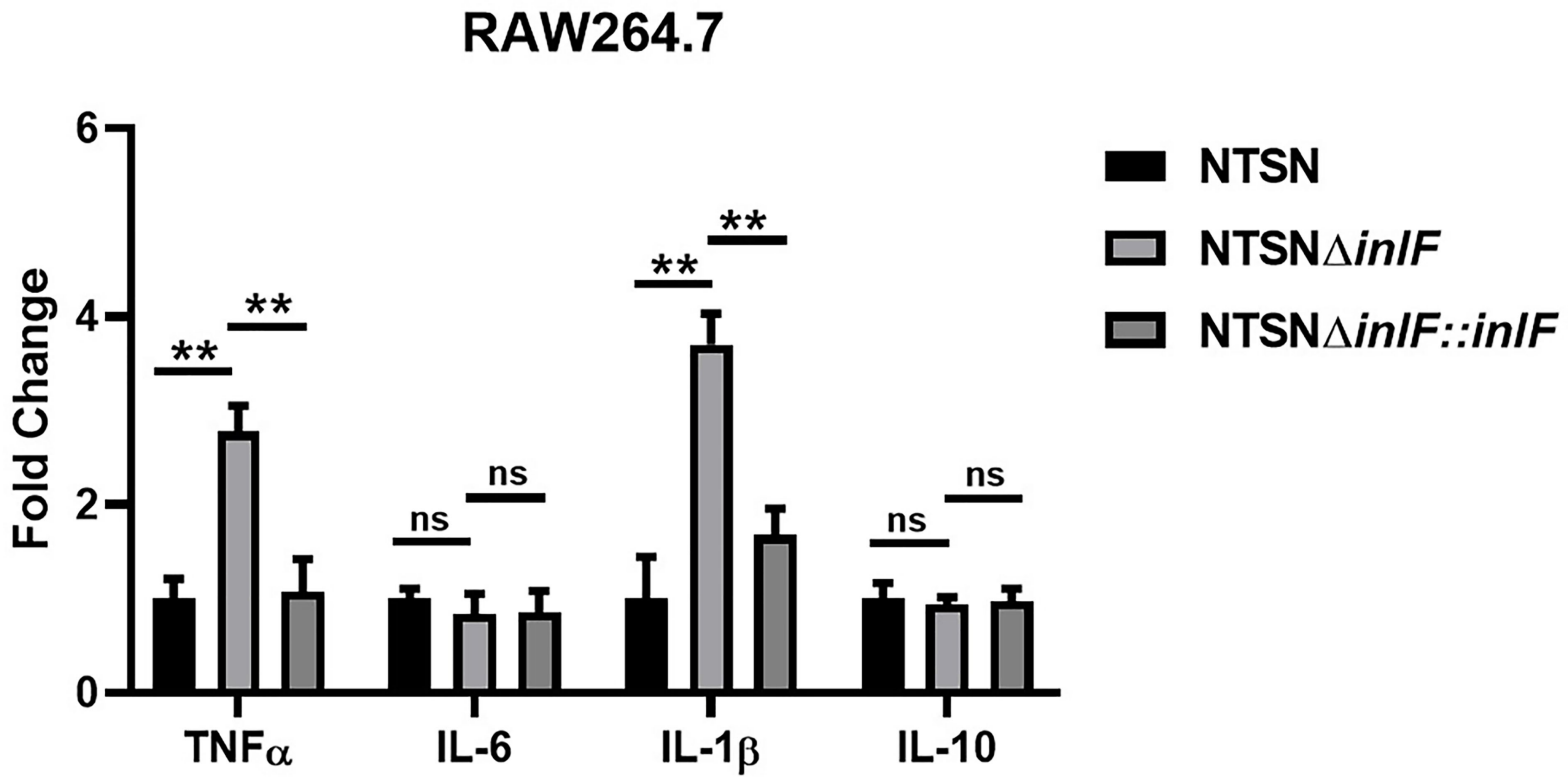
InlF impairs the proinflammatory response in vitro. RAW264.7 cells were infected (MOI = 20) with Lm NTSN NTSNΔ*inlF* and NTSNΔ*inlF::inlF*. The determination was performed 1h of bacteria invasion. Among them, the PBS negative control group is represented by horizontal line bar, NTSN is represented by black and white check bar and the mutant is represented by black dot bar. Values are mean with error bars; n = 3 independent experiments. Statistical analysis was carried out by Tukey’s multiple comparisons test: **P < 0.01, ns, no significance.

RAW264.7, a line of mouse peritoneal macrophages, is a commonly used inflammatory cell model. The interaction between Lm and macrophages RAW264.7 was assessed by examining the expression of inflammatory cytokines IL-10, IL-6, IL-1β, and TNF-α. While there were no statistical differences in the transcriptional expression of IL-10 or IL-6 between Lm NTSNΔ*inlF* and the wild parental and reversion strains (*p >*0.05), the expressions of TNF-α (*p <*0.01) and IL-1β (*p <*0.01) were significantly higher in the mutant strain (**Figure 3**). These results indicated that the deletion of *inlF* triggered a strong upregulation in the expression of pro-inflammatory factors, and that *inlF* may act as a modulator of the anti-inflammatory response during Lm infection *in vitro*.

### Crucial Role of *inlF* in Suppression of the Inflammatory Response

**Figure 4:**
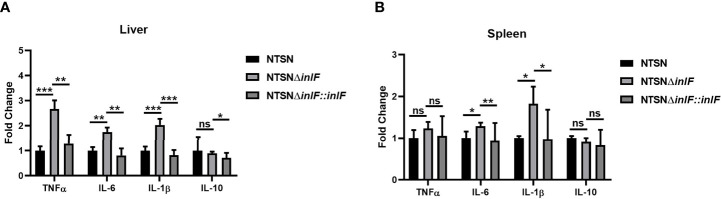
*InlF* modulates the inflammatory process *in vivo*. Mice were inoculated intraperitoneally with Lm NTSN NTSNΔ*inlF* and NTSNΔ*inlF::inlF*, the levels of cytokines in the spleen and liver were measured at 6 h post-infection. qRT-PCR detected the expression of inflammatory cytokines TNF-α, IL-6, IL-1β, and IL-10 in mice liver **(A)** and spleen **(B)**. Among them, the PBS negative control group is represented by horizontal line bar, NTSN is represented by black and white check bar and the mutant is represented by black dot bar. Values are mean with error bars; n = 3 independent experiments. Statistical analysis was carried out by Tukey’s multiple comparisons test: *P < 0.05, **P < 0.01, ***P < 0.001, ns, no significance.

Macrophages are widely distributed in various tissues and function in homeostasis (Zhang X. M. et al., 2019). To understand the role of *inlF* in mediating the Lm defense against host immune responses, we measured the transcriptional expression of inflammatory factors IL-10, IL-6, IL-1β, and TNF-α in the spleen and liver of infected mice. Strikingly, infection with NTSNΔ*inlF* triggered significantly higher production of cytokines IL-6 (*p <*0.05) and IL-1β (*p <*0.05) in the spleen; there was no obvious difference in TNF-α elicited by three Lm strains (*p >*0.05) (**Figure 4A**). Consistently, the transcriptional expression of IL-6 (*p <*0.01) and IL-1β (*p <*0.001) was also significantly higher in the liver of mice infected with NTSNΔ*inlF*. Importantly, following the infection of NTSNΔ*inlF*, there was significantly higher expression of TNF-α in the liver (*p <*0.001) (**Figure 4B**). The above data suggested that *inlF* played a crucial role in modulating the suppression of the pro-inflammatory response *in vivo*.

### Requirement of *inlF* for the Early Colonization of Lm

**Figure 5:**
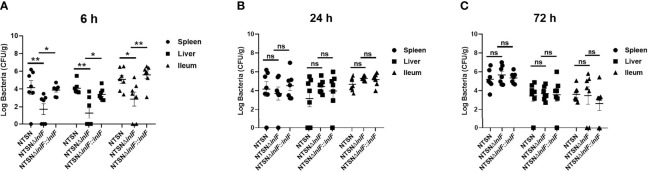
*InlF* contributes to Lm infection during murine listeriosis. Lm (1 × 10^9^ CFU) were intragastrically inoculated to mice (n = 7). Animals were euthanized 6 h **(A)**, 24 h **(B)**, and 72 h **(C)** after infection. The spleen, liver and ileum were recovered, homogenized and plated on BHI or MOX plates. The numbers of bacterium able to colonize the organs are shown. Among them, the PBS negative control group is represented by horizontal line bar, NTSN is represented by black and white check bar and the mutant is represented by black dot bar. Values are mean ± SEM; n = 3 independent experiments. Statistical analysis was carried out by Tukey’s multiple comparisons test: *P < 0.05, **P < 0.01, ns, no significance.

As a facultative intracellular parasite, Lm adheres to and invades intestinal cells with the contribution of internalin proteins, which enable it to break through the intestinal barrier. We compared the colonization abilities of the parental strain and the *inlF* deletion strain following the inoculation of mice *via* the intragastric route. Six hours after inoculation, the bacterial loads in the spleen (*p <*0.01), liver (*p <*0.01), and ileum (*p<<*0.05) were significantly lower in mice infected with NTSNΔ*inlF* compared with the parental and reversion strains (**Figure 5A**). At later time points after infection, 24 h (**Figure 5B**) and 72 h (**Figure 5C**), there were no statistical differences between strains in the bacterial loads in the liver, spleen, and ileum. Taken together, these results indicated that *inlF* was involved in Lm colonization in the early stage of infection.

### Contribution of *inlF* to Lm Pathogenesis

**Figure 6:**
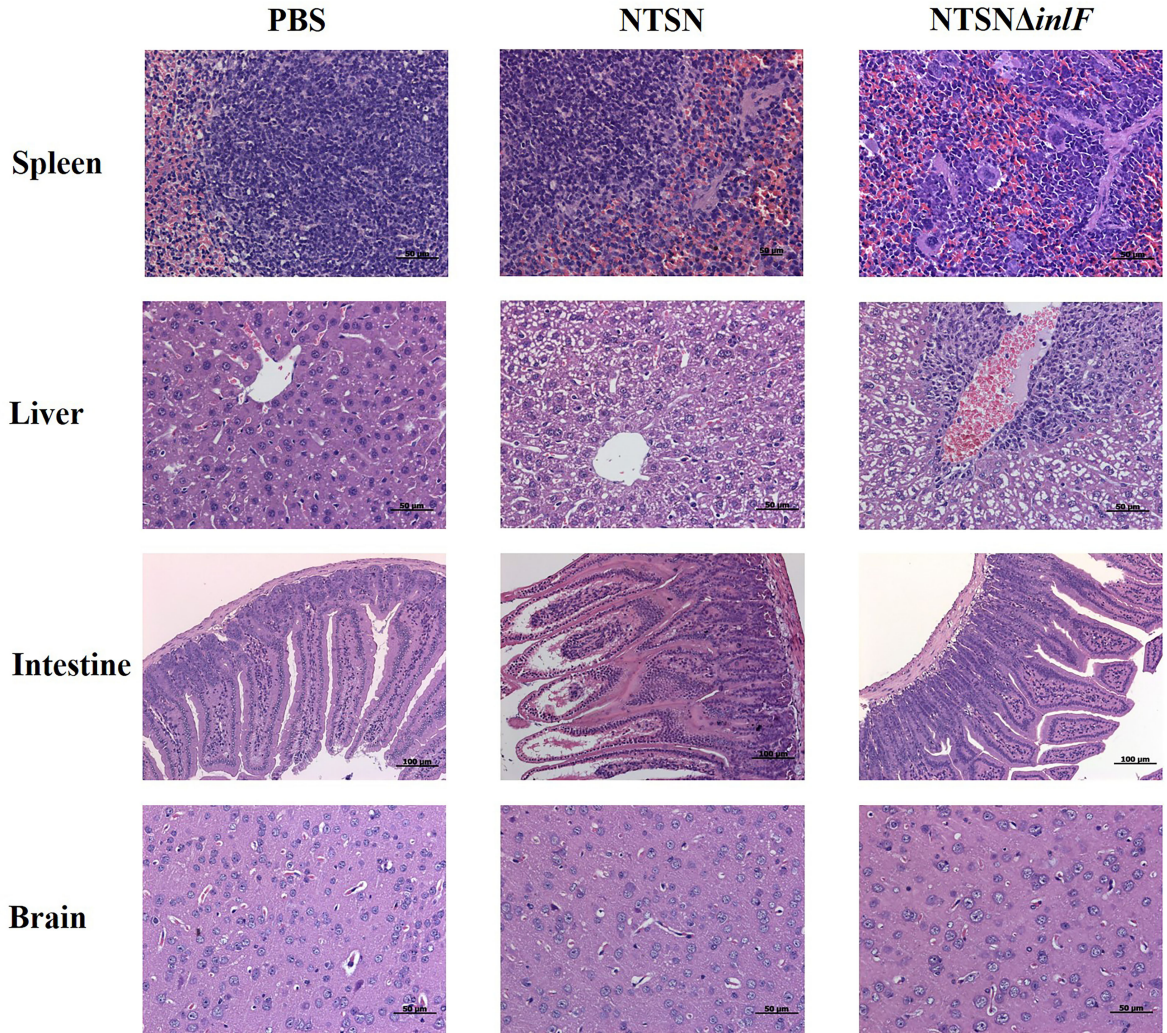
Histopathological section of infected mice organs. Six-week-old female mice were randomly assigned to 3 groups as follows to receive either NTSN or NTSNΔ*inlF* combined with PBS as a control. Experiments were performed at 72 h post-infection. Sections of the spleens, livers, ileum, and brains are shown at the same magnification.

Histologic analysis was performed on tissue sections of mice infected with Lm NTSN or NTSNΔ*inlF* 72 *h* post infection. Compared with a PBS control, NTSN infection led to severe lesions in multiple tissues; spleens exhibited local lesions, necrosis and neutrophil infiltration, some hepatocytes appeared fatty with granular degeneration; there was erosion of the lamina propria of the intestinal mucosa; and the nuclei of nuclei in cells that comprise the intestinal glands of the lower ileum were fragmented or dissolved. Lm NTSNΔ*inlF* caused mild pathologic changes, including proliferation of macrophages in the spleen, neutrophil infiltration, and increased phagocytes in the connective tissue of the liver hilar region. Additionally, there were no obvious pathologic changes in the brains of either of the infected groups, which might be explained by the relatively short infection time (**Figure 6**). These observations result indicated that *inlF* was critical for Lm virulence and contributed to infection and pathogenesis *in vivo* in mice.

## Discussion

Pathogenic Lm has the ability to invade various cell types, and encodes internalin proteins with specific roles in bacterial infection and immunity. Since the first internalin protein (InlA) was identified and functionalized in 1991, 25 internalins containing the LRRs have been successively identified in the Lm strain EGD-e. Surface protein InlF, also a member of the internalin family, was identified in the Lm strain EGD as early as 1997; however, based on *in vitro* and *in vivo* experiments, Dramsi et al. and Senay et al. verified that it did not play a role in the adhesion and invasion of non-phagocytic cells, and was not essential for infecting mice (Dramsi et al., 1997; Senay et al., 2020). Kirchner and colleagues investigated the impact of the ROCK pathway on Lm serovar 1/2a 10403S infection of mammalian cells and found that inhibition of ROCK activity contributed to InlF-mediated invasion and virulence (Kirchner and Higgins, 2008). A recent study verified that *inlF* from intravenous inoculation with Lm 10403S was able to coordinate with host cell-surface vimentin and contribute to Lm invasion of the brain (Ghosh et al., 2018). Previous research on InlF was mainly conducted on evolutionary lineageII strains; however, there is only 78% homology in the amino acid sequence of InlF protein between lineage I and lineageII strains (**Supplementary Figure 3**). Furthermore, because the majority of invasive 4b strains in human outbreaks of listeriosis belonged to lineage I, the contribution of InlF in mediating infection and pathogenesis is not fully understood.

To obtain a more comprehensive understanding the roles of *inlF* in Lm serovar 4b infection, we compared its transcriptional expression with *inlA* and *inlB* during infection of five non-phagocytic cell types and macrophages RAW264.7 and also analyzed its expression in the liver, spleen and brain of infected mice. Unexpectedly, we found that *inlF* was significantly upregulated not only in non-phagocytic cells and macrophages *in vitro*, but also in all three organs *in vivo*. These results suggested that *inlF* played an important role in the interaction between Lm and various types of host cells. The dramatically enhanced colonization capability in the ileum during the early stage of infection in mice, and following subsequent severe pathologic damage to the liver, spleen, and ileum, strongly supports a role for *inlF* in Lm virulence and systemic infection. Authors of previous studies of *inlF* using Lm 4b serovar JF5203 concluded that the lack of *inlF* had no significant effect on infection or growth (Rupp et al., 2017). Senay et al. constructed an *inlF*-deficient 4b strain to assess its role in the infection of multiple cell types and found, that *inlF* was not required for hyper-invasiveness (Senay et al., 2020). By contrast, our infection experiments in mice have demonstrated a role of *inlF* in the virulence and pathogenesis of a serovar 4b strain.

Lm is a facultative intracellular pathogen that can escape from phagocytic vesicles into the host cytoplasm, where it regulates the innate and adaptive immune responses, and decreases host resistance to systemic infection (Stockinger et al., 2004; Opitz et al., 2006; Boneca et al., 2007). Multiple *Listeria*-specific genes are involved in the interference with the innate immune response that occurs during Lm–phagocytic cell interaction. *PdgA* and *OatA*, which are associated with the synthesis of peptidoglycan for the bacterial cell wall, mediate Lm evasion from the innate immune response (Boneca et al., 2007; Aubry et al., 2011), while the internalin family protein InlC dampens the nuclear factor (NF)-кB signaling pathway by targeting the inhibitor of NF-кB, IкB kinase (Gouin et al., 2010). InlH from the serovar 1/2a EGD-e strain has been reported to mediate IL-6 secretion during murine listeriosis. InlF protein contains 13 LRR domains and an LPXTG motif, marking it structurally similar to InlH (Bierne et al., 2007). Whether *inlF* also contribute to evasion of the host immune response remains unclear and deserves further elucidation.

To investigate the potential role of *inlF* in mediating Lm interference with the innate immune response, we constructed an *inlF*-deficient and reverse Lm strain, compared it with the parental strain for effects on cytokines during infection of macrophages and mice. Interestingly, we found that *inlF* did significantly inhibit the production of pro-inflammatory cytokines TNF-α and IL-1β, enhancing the survival of Lm in macrophages *in vitro*. *In vivo*, *inlF* strongly suppressed the production of TNF-α, IL-6, and IL-1β in the liver, as well as IL-6 and IL-1β in the spleen. As the effector cytokines, IL-6, TNFα, and IL-1β are clearly required for initiation of the Th1-type immune response and for direct bacterial killing (Allie et al., 2013; Romagnoli et al., 2017). The suppression of TNF-α and IL-1β production undoubtedly promoted the ability of Lm NTSN to evade the direct bacteria-killing of macrophages, thus contributing to its survival during the early stage of infection. The inhibitory effect of *inlF* on IL-6 *in vitro* and *in vivo* was consistent with the known involvement of *inlH* in immune invasion; however, the obvious difference in invasion ability between *inlF* and *inlH* in RAW264.7 cells suggested that *inlF* may play a more important role in immune evasion (Personnic et al., 2010). Pro-inflammatory cytokines (e.g., IL-6, TNFα) and chemokines (e.g., CXCL2) are upregulated by the activation of the NF-κB pathway upon the host sensing the presence of cytosolic Lm *via* target receptors (D'Orazio, 2019). We deduced that *inlF* may be involved in blocking the NF-κB pathway, thereby downregulating the inflammatory response and contributing to Lm infection and colonization of the host.

Taken together, our results suggest that *inlF* from a serovar 4b strain of Lm plays a crucial role in modulating the innate immune response through interference with the production of multiple pro-inflammatory cytokines, thereby contributing to survival in macrophages and colonization in the early stage of infection. This study has verified that *inlF* as an important virulence factor involved in immune evasion and pathogenesis.

## Data Availability Statement

The original contributions presented in the study are included in the article/**Supplementary Matreial**. Further inquiries can be directed to the corresponding authors.

## Ethics Statement

The animal study was reviewed and approved by the Institutional Animal Ethics Committee of Yangzhou University.

## Author Contributions

YY and XJ designed the experiments. ZL and DZ performed the experiments and analyzed the results. YW and SK were involved in animal experiments. XX and HY participated in experiments related to immune escape. ZL and YY wrote the manuscript. XC and ZP provided a lot of guidance in the design and writing process. All authors contributed to the article and approved the submitted version.

## Funding

This work was supported by the National Key Research and Development Program of China (Nos. 2017YFC1601201), the National Natural Science Foundation of China (No. 31472193), the Jiangsu agricultural science and technology independent innovation funds (CX(21)1004), the Key Research and Development Program (Modern Agriculture) Project of Jiangsu Province (No. BE2017341), the Jiangsu Agricultural Science and Technology Independent Innovation Funds [No. CX(21)1049], and the Priority Academic Development Program of Jiangsu Higher Education Institutions (PAPD).

## Conflict of Interest

The authors declare that the research was conducted in the absence of any commercial or financial relationships that could be construed as a potential conflict of interest.

## Publisher’s Note

All claims expressed in this article are solely those of the authors and do not necessarily represent those of their affiliated organizations, or those of the publisher, the editors and the reviewers. Any product that may be evaluated in this article, or claim that may be made by its manufacturer, is not guaranteed or endorsed by the publisher.
