# Supplementary material for: inlF Enhances Listeria monocytogenes Early-Stage Infection by Inhibiting the Inflammatory Response
Source: Front Cell Infect Microbiol. 2022 Feb 10;11:748461. doi: 10.3389/fcimb.2021.748461 (PMC8866704; doi:10.3389/fcimb.2021.748461)
Supplement: Supplementary file 1 [file DataSheet_1.pdf]

1.1 Supplementary Figures

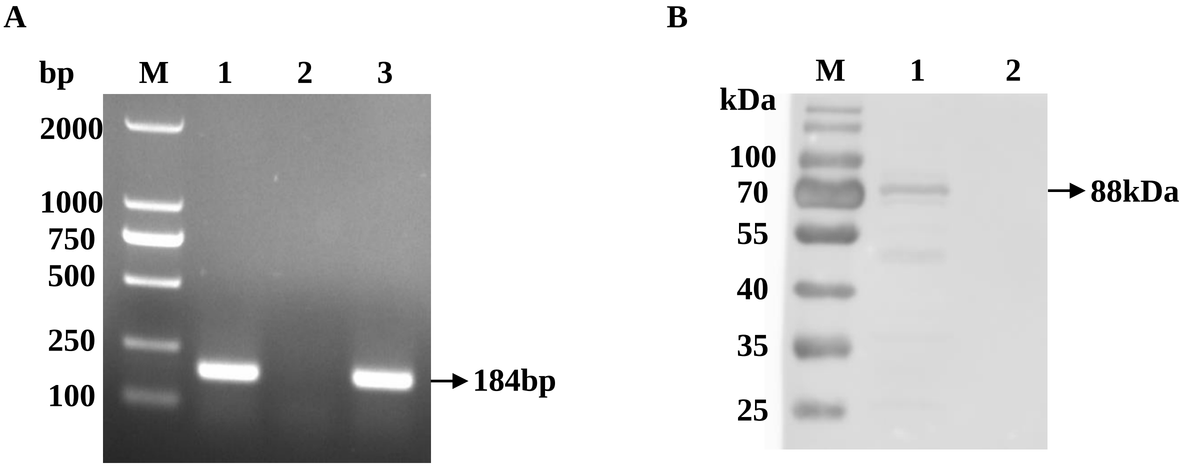

Supplementary Figure 1. Verification of *inlF* gene deletion. (A) RT-PCR analysis to verify the deletion of *inlF*. M: DL2000 DNA marker; 1: PCR products of NTSN cDNA; 2: PCR products of NTSNΔ*inlF* cDNA； 3: PCR products of NTSNΔ*inlF::inlF* cDNA. (B) Western blot analysis of InlF protein expression by Lm NTSN and NTSNΔ*inlF*. M: Prestained protein Ladder; 1: proteins InlF from NTSN; 2: proteins from NTSNΔ*inlF*.

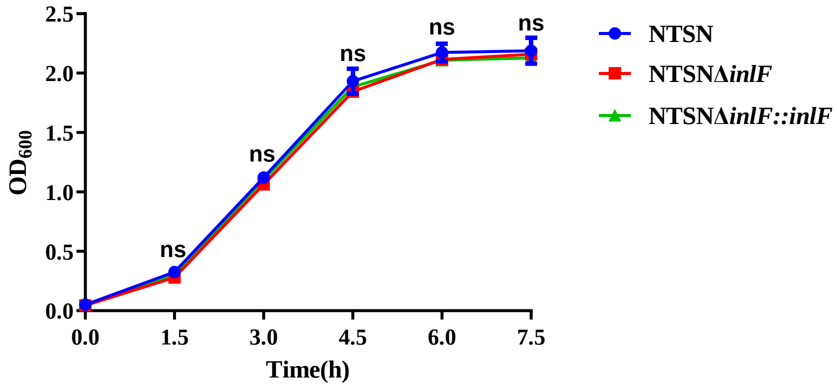

Supplementary Figure 2. The growth curve of Lm. Three initial OD_{600}-identical bacterium were cultured in BHI medium at 37°C. There was no difference among Lm NTSN, NTSNΔ*inlF* and NTSNΔ*inlF::inlF*.

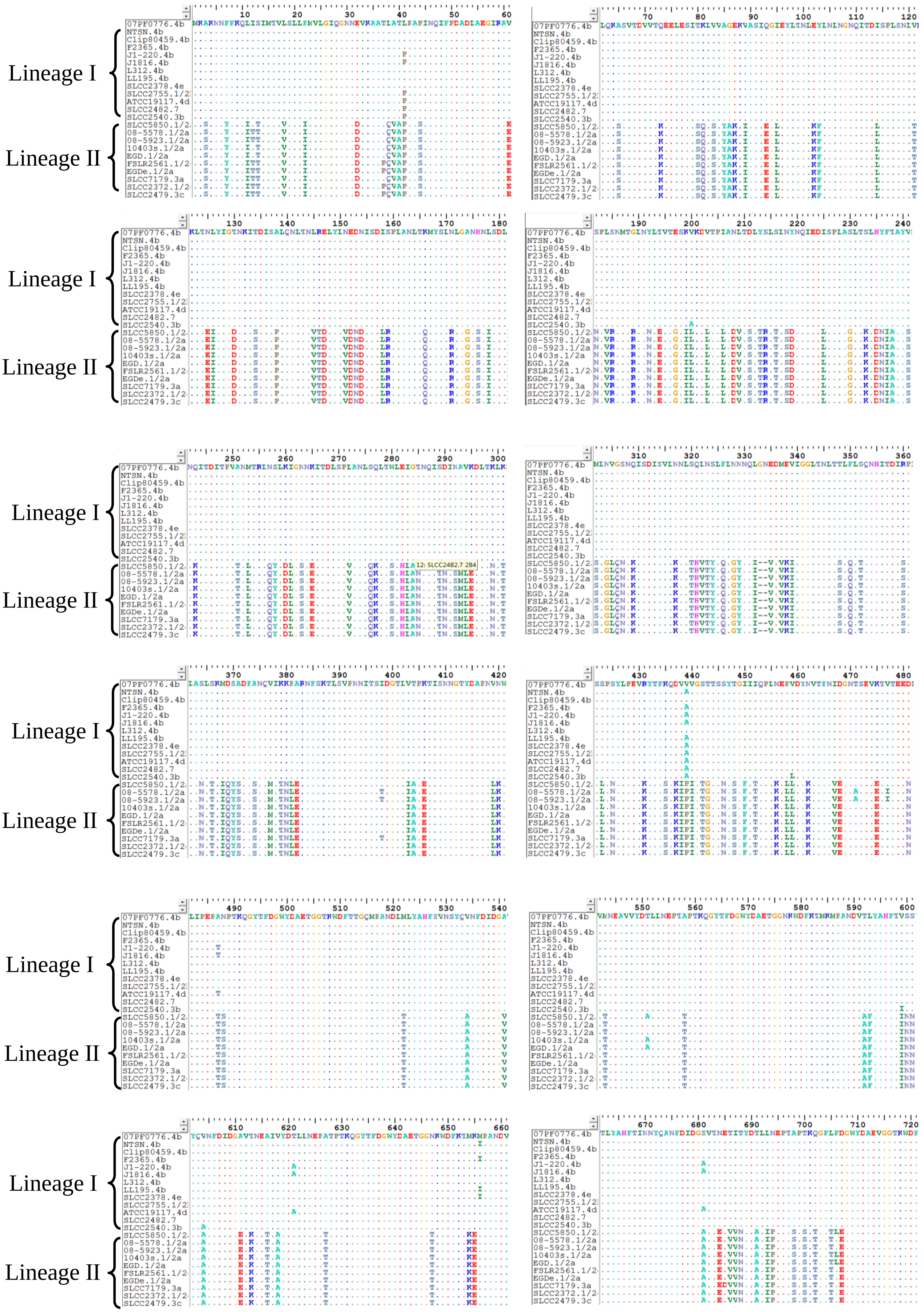
Lineage I
Lineage II

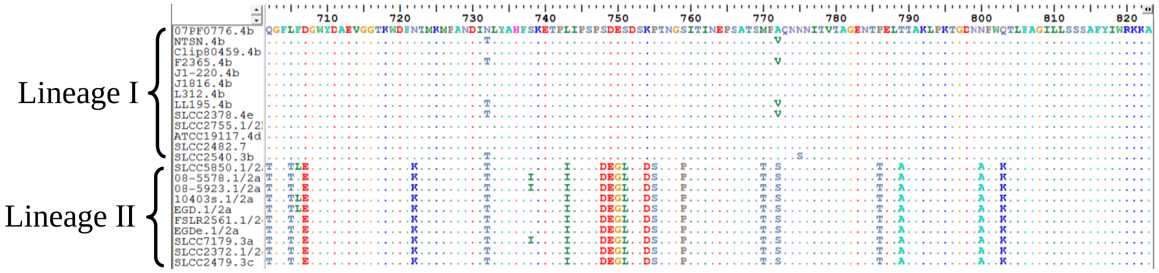

Supplementary Figure 3. Analysis of amino acid sequence alignments of InlF protein in lineage I and II. The amino acid sequences of 23 strains of InlF protein were downloaded from the NCBI database and compared by MEGA5 software. The total length of InlF protein in lineage I was 823 amino acids, and that in lineage II was 821. There were 114 heterotopias in the amino acid sequence of the two lineages, and the homology was 78%.

1.2 Supplementary Tables

Supplementary Table 1. Cell lines used in this study. The top five are nonphagocytes, and the last one is macrophage.

Cells	Relevant characteristics	Reference/Source
Caco-2	Human epithelial colorectal adenocarcinoma cell line	ATCC CRL-2102
NIH/3T3	Mouse embryonic fibroblast cell	ATCC CRL-1658
BRL-3A	Rat liver cell line	ATCC CRL-1442
Hep G2	Human liver cancer cell line	ATCC HB-8065
HBMEC	Human brain microvascular endothelial cell	Cell Systems Corporation ACBRI376
Hela	Human cervical cancer cell	ATCC CCL-2

RAW 264.7	mouse leukemia cells of monocyte macrophage	ATCC TIB-71

Supplementary Table 2. Biochemical identification of Lm. 43 physiological and biochemical reactions of Lm were detected by automatic microorganism identification instrument. There was no difference among Lm NTSN and NTSNΔ*inlF*.

Biochemical	NTSN	NTSNΔ*inlF*	NTSNΔ*inlF::inlF*
AMY	+	+	+
PIPLC	+	+	+
dXYL	-	-	-
ADH1	-	-	-
BGAL	-	-	-
AGLU	+	+	+
APPA	-	-	-
CDEX	+	+	+
PyrA	-	-	-
BGUR	-	-	-
AlaA	-	-	-
TyrA	+	+	+
dSOR	-	-	-
URE	-	-	-
POLYB	+	+	+
dGAL	-	-	-
dMAN	-	-	-
dMNE	+	+	+
MBdG	+	+	+
PUL	-	-	-
dTRE	+	+	+
ADH2s	-	-	-
AspA	-	-	-
BGAR	-	-	-
AMAN	+	+	+

PHOS	-	-	-
LeuA	+	+	+
ProA	-	-	-
BGURr	-	-	-
AGAL	-	-	-
dRIB	-	-	-
ILATk	-	-	-
LAC	+	+	+
NAG	+	+	+
dMAL	+	+	+
BACI	+	+	+
NOVO	+	+	+
NC6.5	+	+	+
Draf	-	-	-
O129R	+	+	+
SAL	+	+	+
SAC	+	+	+
OPTO	+	+	+

Supplementary Table 3. Primers for quantitative real-time PCR analysis.

Primer code	Primers Sequences (5'-3')
InlA1	ACACTACACCACCTTCCG
InlA2	GCATTATCGCTATCACCA
InlB1	GAGCGTTAGCAGGACTTA
InlB2	TCGCCATCATCACTTATT
InlF1	CCGCTAAATGAGCCAGTA
InlF2	GCCCAGTTGTAAAGTCCC
Gyrb1	AGACGCTATTGATGCCGATGA
Gyrb2	GTATTGCGCGTTGTCTTCGA
TNF-α-F	TCTCATTCCTGCTTGTGG

TNF-α-R	ACTTGGTGGTTTGCTACGA
IL-1β-F	GCCACCTTTTGACAGTGATG
IL-1β-R	TGATGTGCTGCTGCGAGA
IL-10-F	ACCTGGTAGAAGTGATGCC
IL-10-R	GACACCTTGGTCTTGGAG
IL-6-F	TACCACTCCCAACAGACC
IL-6-R	CATTTCCACGATTTCCCAGA
GAPDH-F	CAAATTCAACGGCACAGTCA
GAPDH-R	TTAGTGGGGTCTCGCTCC

Supplementary Table 4. Primers for mutant and complemental strain’s construction.

Primer code	Primers sequences (5’-3’)[a]	Digestion sites	Size of PCR product (bp)
*inlF*a-F	CTGTCGACAAAGTGGTTCGGGAAATCCCG	*Sal*I	501
*inlF*a-R	GGATATACTGGGTTTTTAATTTAGTACCTTCCTTCAAC		
*inlF*b-F	GTTGAAGGAAGGTACTAAATTAAAAAACCCAGTATATCC		702
*inlF*b-R	GCGGTACCAAACAAAAGAAAAAATCG	*Kpn*I	
*inlF*W1	AGTTAGCTTGGATTTTAACGCT		4071/1599
*inlF*W2	GGGTGAAATTGACTTAACCAAAACT		
inlFc-F	TTGTAAAACGACGGCCAGTGAATTCAGGCTTATCCAATCCACT	EcoRI	2195
*inlF*c-R	TTCTTCTGTTACAGTTTTTACTGCGTTTGTATTGCCGTCTAT		
inlFd-F	ATAGACGGCAATACAAACGCAGTAAAAACTGTAACAGAAGAA		1956
*inlF*d-R	TGCATGCCTGCAGGTCGACTCTAGAGCGGGTGAAATTGACTTA	XbaI	
*inlF*W3	CGGATTGACGGTAGAAT		4460/1990
*inlF*W4	AAATGTCGCCGATAAAC		

[a]The inlFa-F and inlFb-R primers contain homologous fragments of the pGEM-T vector. The restriction endonuclease used are marked with the following lines.
